# Longitudinal associations between circulating interleukin-6 and C-reactive protein in childhood, and eating disorders and disordered eating in adolescence

**DOI:** 10.1016/j.bbi.2020.07.040

**Published:** 2020-10

**Authors:** Francesca Solmi, Cynthia M. Bulik, Bianca L. De Stavola, Christina Dalman, Golam M. Khandaker, Glyn Lewis

**Affiliations:** a Division of Psychiatry, UCL, London, UK; b Department of Psychiatry, University of North Carolina at Chapel Hill, Chapel Hill, USA; c Department of Nutrition, University of North Carolina at Chapel Hill, Chapel Hill, USA; d Department of Medical Epidemiology and Biostatistics, Karolinska Institutet, Stockholm, Sweden; e Great Ormond Street Institute of Child Health, UCL, London UK; f University of Cambridge, Cambridge, UK; g Cambridgeshire and Peterborough NHS Foundation Trust, Cambridge, UK

**Keywords:** Eating disorders, Inflammation, IL6, CRP, ALSPAC, Cohort study

## Abstract

**Objective:**

Few studies have explored the association between inflammation and eating disorders and none used a longitudinal design. We investigated the association between serum-levels of interleukin 6 (IL-6) and C-reactive protein (CRP) measured in childhood and eating disorders and related behaviours and cognitions in adolescence in a large general population sample.

**Methods:**

We used data from the Avon Longitudinal Study of Parents and Children (ALSPAC). Our exposures were thirds of IL6 and CRP derived from serum measurements taken at age nine years, and outcomes were eating disorder diagnoses and self-reported disordered eating behaviours at ages 14, 16, and 18 years. We used univariable and multivariable multilevel logistic regression models adjusting for a number of potential confounders, including sex, fat mass, and pre-existing mental health difficulties.

**Results:**

Our sample included 3480 children. Those in the top third of CRP had lower odds of binge eating (odds ratio(OR):0.62, 95% confidence interval (CI):0.39,1.00,p “equals” 0.05) and fasting (OR:0.63, 95% CI:0.38,1.07,p “equals” 0.09) after adjustment for confounders. We also observed weak associations of comparable magnitude for purging, anorexia nervosa, and bulimia nervosa. We did not find any associations between levels of IL6 and any of the outcomes under study.

**Conclusions:**

There was little evidence of an association between CRP and IL-6 and adolescent eating disorder outcomes. The inverse association observed between CRP and binge eating was unexpected, so caution is needed when interpreting it. One possible explanation is that higher CRP levels could have a protective role for disordered eating by affecting appetitive traits.

## Highlights

• Levels of IL6 at 9 years were not associated with eating disorders at 14, 16, and 18.
• Lower CRP levels at 9 were associated with greater disordered eating in adolescence.
• The associations we observed were weak and residual confounding cannot be excluded.

## 1 Introduction

It is proposed that low-grade systemic inflammation may play a role in the pathogenesis of a number of psychiatric disorders. Cross-sectional studies have shown that individuals with depression and anxiety commonly display altered immunological profiles (Costello et al., 2019, Osimo et al., 2019). Elevated serum concentrations of pro-inflammatory cytokines, such as Interleukin-6 (IL-6) and C-Reactive Protein (CRP), are also prospectively associated with depression (Khandaker et al., 2014, Valkanova et al., 2013), lending plausibility to a putative causal association. In addition, Mendelian randomization studies support potential causal associations between IL-6, CRP and depression (Khandaker et al., 2019, 2018). Eating disorders, related disordered eating behaviours and cognitions are commonly comorbid with anxiety and depression (Konttinen et al., 2019, Micali et al., 2015b, Solmi et al., 2014). However, despite the burgeoning inflammation literature, only a limited number of studies have investigated their association with pro-inflammatory cytokines (Brambilla, 2001, Brambilla et al., 2001, Caroleo et al., 2019, Dalton et al., 2018a, Dalton et al., 2018b, Germain et al., 2016, Holden and Pakula, 1996, Kahl et al., 2004, Nakai et al., 2001, Pomeroy et al., 1994, Raymond et al., 2000, Shank et al., 2016, Solmi et al., 2015).

To the best of our knowledge, none of these studies used longitudinal designs measuring cytokines prior to the onset of eating disorders, which are necessary to investigate whether inflammation is a potential risk factor for these disorders. The majority of existing studies used case-control designs in clinical populations. These designs are susceptible to selection bias and reverse causation. Existing evidence suggests that women with anorexia nervosa have higher levels of pro-inflammatory cytokines IL-6, and lower levels of CRP during acute illness (Dalton et al., 2018a, Solmi et al., 2015). However, these biomarkers return within normal ranges with weight restoration (Dalton et al., 2018a, Solmi et al., 2015) indicating that reverse causation is indeed plausible. Research on other eating disorders (e.g., bulimia nervosa and binge-eating disorder) and disordered eating is scant, and results from these studies are mixed, with limited evidence of cross-sectional associations between binge eating and higher levels of CRP (Shank et al., 2016, Succurro et al., 2015).

The aim of this study is to investigate whether serum levels of IL-6 and CRP at age nine years are associated with eating disorder diagnoses, disordered eating behaviours and cognitions during adolescence using a large UK general population prospective birth cohort.

## 2 Material and methods

### 2.1 Sample

We used data from the Avon Longitudinal Study of Parents and Children (ALSPAC), a birth cohort that recruited 14,541 pregnancies with an expected delivery date falling between 1st April 1991 and 31st December 1992 in the region of Avon. These pregnancies resulted in 14,062 live births, with 13,988 children alive at one year of age (Boyd et al., 2013, Fraser et al., 2013). The ALSPAC Law and Ethics committee and the Local Research Ethics committees gave ethical approval for the study. The study website (www.bristol.ac.uk/alspac) provides more information on the sample and contains details of all the data that is available through a fully searchable data dictionary: http://www.bris.ac.uk/alspac/researchers/data-access/data-dictionary/.

In this study, we included children who were in the core ALSPAC sample and alive at one year, and who had data available on both exposures, and outcomes (described below in sections 2.2 and 2.3). In case of twins, given their shared genetic and environmental exposures, we retained one child at random to avoid biased inferences.

### 2.2 Outcomes

Adolescents reported on disordered eating behaviours occurring in the previous 12 months at approximately age 14, 16, and 18 years via postal questionnaires using a number of questions from the Youth Risk Behavior Surveillance System (Brener et al., 1995), which have been previously used in this sample (Bould et al., 2018, Micali et al., 2015a, Solmi et al., 2018). These were: fasting for weight loss for at least 24 hours; purging (self-induced vomiting or laxative use) for weight loss; and binge-eating (eating large amount of food in a short period of time with a sense of loss of control). We considered these behaviours present if they had occurred at least once a month and used them individually as outcomes. Adolescents also reported whether they had dieted for at least one month and up to a year continuously. We additionally created a variable indicating whether any of these behaviours were present, in order to increase statistical power of the analyses. Body Mass Index (BMI) was measured at clinics assessment that took place when adolescents were 13.5, 15.5, and 17.5 years old and supplemented with self-reported measurements when objective measures were not available as these are highly correlated in this sample (r = 0.89). As recommended in case of adolescents, we used age- and sex-standardised BMI measurements (Cole et al., 2007, Cole et al., 2000). From these measures, we further derived eating disorder diagnoses of anorexia nervosa, bulimia nervosa, and binge-eating disorder using DSM-5 criteria. Although eating disorder diagnoses are uncommon and therefore likely to result in underpowered statistical analyses, we included them as outcomes to explore whether the associations appeared to be consistent with those observed for the more broadly defined behaviours. At the age 14 years follow up, adolescents also reported on two cognitive dimensions of eating disorders (body dissatisfaction and weight and shape concerns). Body dissatisfaction was measured using the Body Dissatisfaction Scale (Stice, 2001), weight and shape concerns with three questions from the McKnight Risk Factor Survey (Shisslak et al., 1999). In **eMethods 1**, we describe how we derived these outcomes in detail.

### 2.3 Exposure

Serum-levels of IL-6 and CRP were obtained from non-fasting blood samples when the children were on average 9.9 (standard deviation: 0.32) years of age during clinical assessments. More details on these measurements are available in previous publications (Khandaker et al., 2014). We restricted analyses to participants who had a CRP count < 10 mg/L, as values above this threshold might indicate active infection (Pepys and Hirschfield, 2003), and used standardised values for ease of interpretation. We used thirds of IL-6 and CRP to define equal size groups as main exposures to allow for non-linear associations and, as supplemental analyses also used continuous indicators further adding a quadratic term to explore non-linear associations.

### 2.4 Confounders

**Fig. 1:**
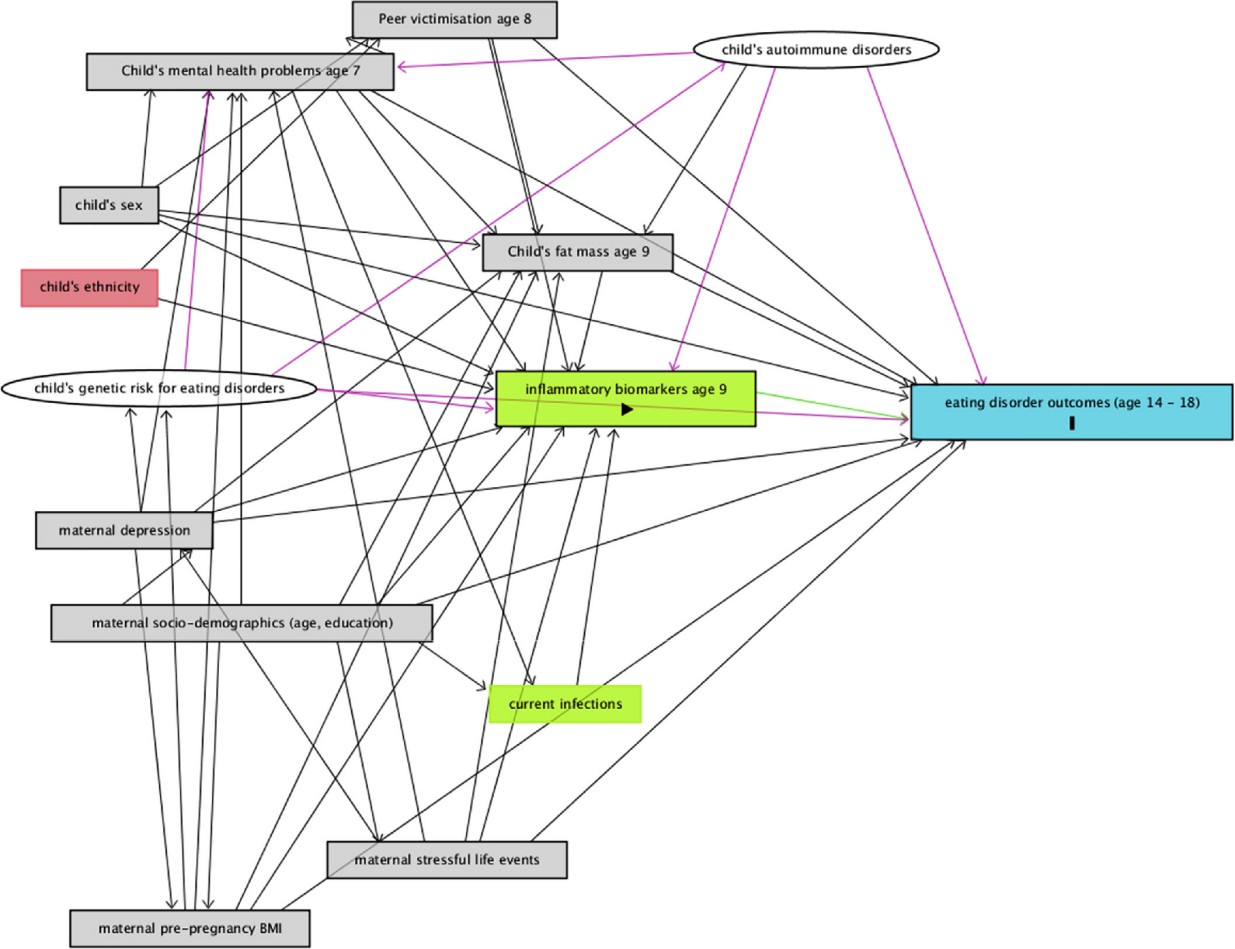
Direct Acyclic Graph (DAG) outlining our causal assumptions. The green square with triangle shape represents our exposures, whereas the blue square with the rectangle represents our outcomes. Empty green squares represent ancestors of the exposure (i.e. factors we assumed to cause the exposure, but not the outcome). Based on our causal assumptions, in order to calculate the total effect of inflammatory biomarkers IL-6 and CRP on the outcome, it was necessary to adjust our models for: child’s sex, genetic risk for eating disorders, autoimmune disorders, fat mass at age nine years, mental health difficulties at age seven years, exposure to bullying at age eight as well as maternal age, highest education (as a proxy for socio-economic position), depression, and stressful life events. Grey squares represent confounders which we could adjust for, as this information is available in the ALSPAC dataset, while white circles represent variables which we were not able to observe in the data. We discuss implication of not adjusting for these variables in the limitations section. Adjustment for child’s ethnicity, in red square, was not required based on our assumptions. (For interpretation of the references to colour in this figure legend, the reader is referred to the web version of this article.)

In order to estimate the total effect of IL6 and CRP on our outcomes, we adjusted our analyses for a pre-specified set of confounders (i.e., factors which we hypothesised could cause both the exposure and the outcome, and thus represent alternative explanations for any observed associations) after defining our causal assumptions using Direct Acyclic Graphs (Fig. 1). These hypothesised associations were informed by previous literature. These included child’s: sex (male/female), total fat mass at the time of exposure measurement (age nine years), mental health problems at age seven years (parentally-reported using the Strength and Difficulties Questionnaire (SDQ) (Goodman, 1997)), and child-reported peer victimization at age eight years. Childhood BMI is longitudinally associated with greater disordered eating (Reed et al., 2017). To account for this, we adjusted our analyses for fat mass as a proxy for BMI, given the strong correlation between pro-inflammatory cytokines and fat tissue and the known limitations of BMI as a measurement of body composition (Dencker et al., 2007, Freedman et al., 2005). Mental health difficulties (Flouri et al., 2020b) and peer victimisation (Takizawa et al., 2015) in childhood are associated with raised inflammatory profiles and have been associated with disordered eating (Lee and Vaillancourt, 2018, Sonneville et al., 2015).

Our causal assumptions also required adjustment for maternal age and highest education at birth of study child (compulsory/non-compulsory) as proxies for socio-economic position, depressive symptoms (self-reported at 32 weeks of gestation using the Edinburgh Postnatal Depression Scale (Murray and Carothers, 1990)), pre-pregnancy BMI, and maternal reported stressful life events (self-reported at 32 weeks of gestation), as proxy for stressful home environment. All of these factors have been associated with increased levels of inflammation in the offspring (Flouri et al., 2020a, Godfrey et al., 2017, Milaniak and Jaffee, 2019, Plant et al., 2016) as well as greater odds of disordered eating and eating disorders (Bould et al., 2015, Larsen et al., 2018, Micali et al., 2018).

As shown in the DAG, we also hypothesized that genetic risk for the outcomes, and existing autoimmune diseases (Raevuori et al., 2014, Zerwas et al., 2017) in the child could be confounders of the association between the exposures and the outcomes. However, we were unable to measure these as, with the exception of anorexia nervosa (Watson et al., 2019), no adequately powered GWAS of disordered eating behaviours currently exists (Boraska et al., 2012, Wade et al., 2013) and autoimmune diseases are rare in children (Cooper and Stroehla, 2003). More details on how these factors were measured in ALSPAC is provided in eMethods 2.

### 2.5 Data analysis

We described the sample characteristics using proportions with percentages and median with interquartile ranges, given the skewed nature of the exposures (eFigs. 2–3). We tabulated the prevalence of disordered eating behaviours in our sample (as previously defined) and among all participants who responded at 14, 16, and 18 years, to explore whether the two were comparable.

We investigated the association between our exposures (measured both as categorical and as continuous exposures) and outcomes at 14, 16, and 18 years using multi-level univariable and multivariable logistic regression models accounting for repeated measurements within participants. We ran three models: a univariable regression including only exposure, outcome, and an indicator of age at outcome measurement (14, 16, and 18 years) and two multivariable ones. In multivariable model one, we progressively adjusted for child’s sex and fat mass. In multivariable model two, we included all other child- and maternal-level confounders. We additionally fitted this model with an interaction term between exposure and age to investigate the presence of age-specific associations.

We tested the association between exposures and eating behaviours and cognitions at age 14 using three linear regression models. Univariable model, only included the exposure, multivariable model one progressively adjusted for sex and fat mass, and multivariable model two additionally including all other confounders.

We imputed missing confounder and outcome data in children with complete exposure and disordered eating behaviours reported at, at least, one follow-up point using multiple imputation with chained equations and 50 imputations (eMethods 3). We also ran all models using complete cases. We used Stata 15 to conduct all analyses (StataCorp, 2017). The protocol for this study was pre-registered and is available at: https://osf.io/2pz59/.

## 3 Results

### 3.1 Sample and missing data

**Fig. 2:**
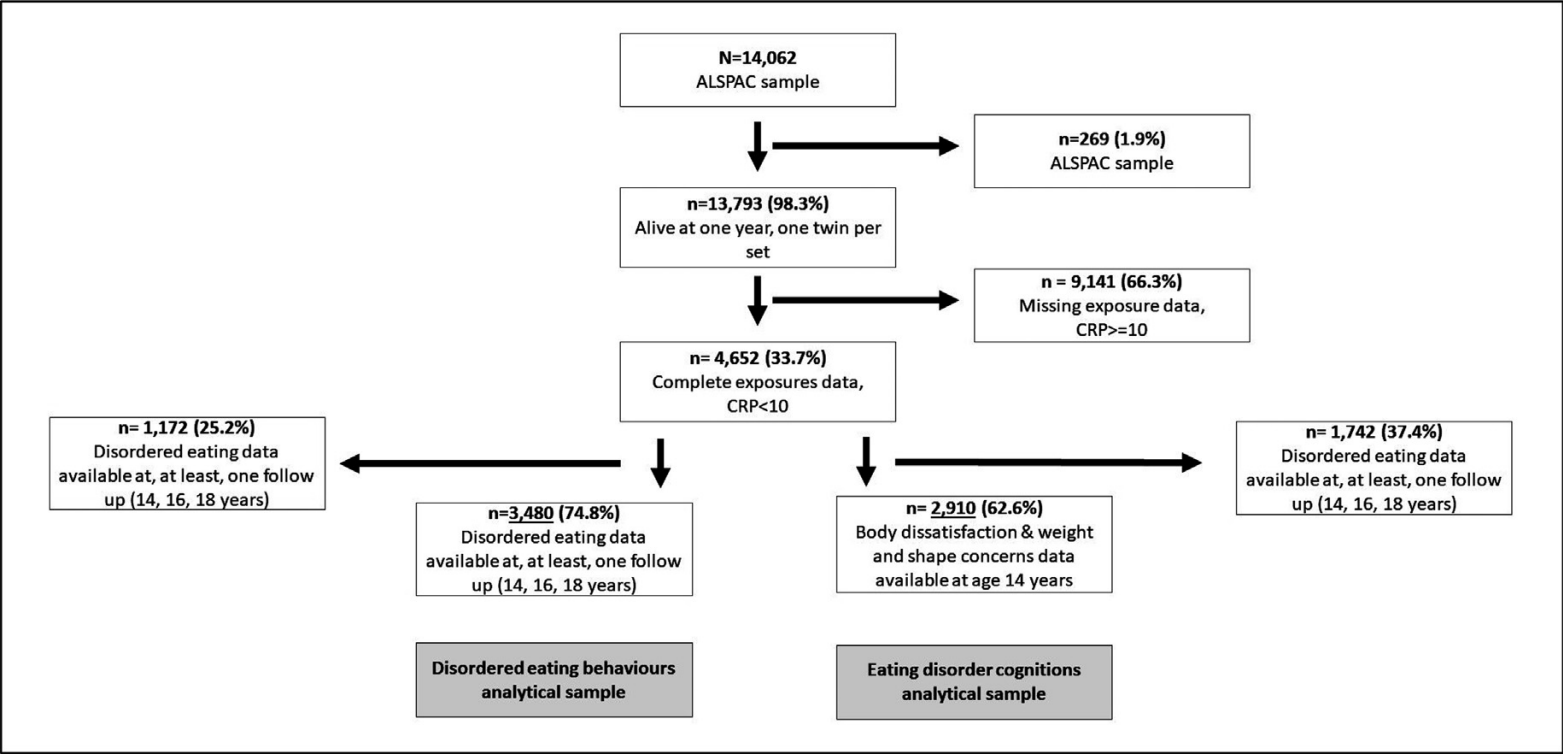
Flow chart of study participation.

Fig. 2 shows a flowchart of study participation. At one year, 13,787 children in the core ALSPAC sample were alive. Of these, 7205 (52.2%) attended clinic assessments at age 9 years, and 4652 (33.7%) had complete data on both exposures, from blood samples.

Children missing exposure data were more likely to be males, to have had greater mental health difficulties at seven years of age, or having been bullied at age eight years. They were also more likely to have younger and less educated mothers, who had greater depressive symptoms. Children who attended the clinic assessment at age nine years, but did not have data on blood sample had similar characteristics with the exception that they were more likely to be girls (see eTable1).

Among those with complete exposure data, 2910 (62.6%) also had data on weight and shape concerns, and body dissatisfaction scales at age 14 years, and 3480 (74.8%) had data on disordered eating behaviours at, at least, one time point in adolescence. IL-6 and CRP were not associated with missing outcome data. However, males, children with mental health difficulties (indexed by above-threshold scores on the SDQ), and those who had been bullied were more likely to have missing outcome data. Greater maternal depressive symptoms, maternal BMI, and younger age at child birth were also associated with greater attrition (eTable 2).

Table 1 presents the characteristics of children with complete exposure data. Median levels of both exposures were higher in girls, and in children with greater fat mass, who had been bullied, and whose mother had higher BMI. Median levels of IL-6 were also higher in children whose families experienced more stressful life events, who had younger mothers, and who had internalising or externalising symptoms.

**Table 1:** Characteristics of participants with complete exposure and median levels (with inter-quartile ranges) of IL-6 and CRP across confounders. (n = 4652).

	N (%)	IL-6 Median (IQR)	CRP Median (IQR)
	4,652	0.80 (0.49,1.38)	0.21 (0.11, 0.52)
**Sex**			
*Male*	2,352 (50.6%)	0.70 (0.45, 1.19)	0.16 (0.10, 0.39)
*Female*	2,300 (49.4%)	0.90 (0.56, 1.53)	0.28 (0.14, 0.67)
**Tertiles of total fat mass**			
*1st tertile (lowest)*	1,540 (34.7%)	0.67 (0.42, 1.13)	0.13 (0.09, 0.25)
*2nd tertile*	1,488 (33.5%)	0.73 (0.46, 1.23)	0.20 (0.12, 0.40)
*3rd tertile (highest)*	1.411 (31.8%)	1.07 (0.68, 1.69)	0.48 (0.24, 1.03)
**Child internalising/externalising problems age 7 years (SDQ)**			
*Absent*	2,328 (96.9%)	0.78 (0.49, 1.36)	0.21 (0.11, 0.51)
*Present*	140 (4.1%)	0.83 (0.54, 1.43)	0.17 (0.11, 0.42)
**Maternal education**			
*Compulsory*	2,512 (55.9%)	0.82 (0.51, 1.40)	0.22 (0.12, 0.54)
*Non-compulsory*	1,984 (44.1%)	0.78 (0.47, 1.35)	0.20 (0.11, 0.49)
**Maternal age**			
*15*–*19*	83 (1.8%)	0.98 (0.59, 1.43)	0.21 (0.12, 0.43)
*20*–*25*	902 (19.4%)	0.86 (0.54, 1.43)	0.24 (0.12, 0.60)
*26*–*35*	3,281 (70.5%)	0.78 (0.49, 1.39)	0.21 (0.11, 0.51)
*36*–*44*	386 (8.3%)	0.78 (0.49, 1.19)	0.19 (0.11, 0.46)
**Maternal pre-pregnancy BMI**			
*Underweight*	185 (4.4%)	0.77 (0.46, 1.32)	0.19 (0.11, 0.40)
*Normal weight*	3,214 (76.0%)	0.77 (0.48, 1.32)	0.20 (0.11, 0.48)
*Overweight*	618 (14.6%)	0.87 (0.57, 1.54)	0.26 (0.13, 0.70)
*Obese*	211 (5.0%)	1.00 (0.57, 1.69)	0.33 (0.13, 0.84)
**Maternal depression**			
*No*	3,807 (89.4%)	0.79 (0.50, 1.37)	0.21 (0.12, 0.52)
*Yes*	452 (10.6%)	0.81 (0.50, 1.37)	0.19 (0.12, 0.52)
**Peer victimization at 8 years**			
*No*	2,645 (67.1%)	0.76 (0.48, 1.34)	0.21 (0.12, 0.54)
*Yes*	1,296 (32.9%)	0.84 (0.52, 1.47)	0.22 (0.11, 0.59)
**Tertiles of Stressful Life Events score**			
*1st tertile (lowest)*	1,614 (37.9%)	0.77 (0.49, 1.34)	0.23 (0.12, 0.54)
*2nd tertile*	1,501 (35.3%)	0.79 (0.49, 1.34)	0.20 (0.11, 0.51)
*3rd tertile (highest)*	1,137 (26.7%)	0.82 (0.55, 1.42)	0.21 (0.12, 0.51)

**List of abbreviations:** CRP = C-Reactive Protein, IL-6 = Interleukin-6, IQR = interquartile range, SDQ = strengths and difficulties questionnaire.

### 3.2 Prevalence of eating disorder diagnoses and disordered eating behaviors

At all ages, disordered eating behaviours and eating disorder diagnoses were uncommon, though their prevalence increased across adolescence. The prevalence of the outcomes in our analytical sample was comparable to that in the overall sample (Table 2).

**Table 2:** Frequency of disordered eating behaviors and diagnoses among those with complete exposure and at least two outcome measurements available and in each wave of data collection overall.

	Disordered eating behaviors (occurring at least monthly)					Eating Disorder diagnoses		
	Any n(%)	Dieting n(%)	Fasting n(%)	Binge eating n(%)	Purging n(%)	AN n(%)	BN n(%)	BED n(%)
**Prevalence among children included in our sample (n = 3,480)**								
**Age 14**	224 (7.47%)	118 (3.94%)	69 (2.30%)	62 (2.07%)	18 (0.60%)	5 (0.18%)	11 (0.37%)	14 (0.47%)
N = 2,998								
**Age 16**	374 (14.7%)	156 (6.13%)	145 (5.74%)	146 (5.74%)	87 (3.42%)	20 (0.86%)	23 (0.90%)	22 (0.86%)
N = 2,545								
**Age 18**	313 (18.21%)	130 (7.56%)	92 (5.35%)	143 (8.32%)	76 (4.42%)	13 (0.82%)	29 (1.69%)	23 (1.34%)
N = 1,719								

**Prevalence among children with data available at each follow-up point**								
**Age 14**	448 (7.98%)	226 (4.03%)	154 (2.74%)	123 (2.19%)	37 (0.66%)	10 (0.21%)	25 (0.45%)	25 (0.45%)
N = 5,611								
**Age 16**	740 (15.82%)	310 (6.63%)	280 (5.99%)	281 (6.01%)	179 (3.83%)	34 (0.85%)	48 (1.03%)	44 (0.94%)
N = 4,678								
**Age 18**	585 (18.65%)	261 (8.31%)	172 (5.48%)	256 (8.15%)	147 (4.68%)	20 (0.73%)	49 (1.56%)	46 (1.46%)
N = 3,140								

### 3.3 Associations between IL-6 and CRP at age nine and disordered eating during adolescence

As shown in Table 3, in univariable models, children in the middle and top third of IL-6 and CRP had greater odds of any disordered eating, and in particular fasting and dieting in adolescence. We observed similar associations for purging and binge eating, although these associations were weak. Adjustment for child’s sex and fat mass in model one, reduced the odds ratios across all outcomes. In this model, there was no evidence of an association between IL-6 and any of the outcomes across adolescence. However, there was some evidence that children in the top third of CRP had lower odds of any disordered eating (OR [odds ratio]: 0.71, 95% CI [confidence interval]: 0.51 to 0.98, p = 0.03) binge eating (OR: 0.61, 95% CI: 0.38 to 1.00, p = 0.05) at ages 14 to 18 years. We also observed associations of similar magnitude for fasting (OR: 0.65, 95%CI: 0.38 to 1.10, p = 0.10) and purging (OR: 0.63, 95%CI: 0.32 to 1.26, p = 0.19) in adolescence, although for these outcomes evidence of an association was weaker, particularly for purging. Further inclusion of all other confounders in model 2 resulted in minimal changes to these associations (any disordered eating OR: 0.70, 95%CI: 0.51 to 0.97, p = 0.03, binge eating OR: 0.62, 95%CI: 0.39 to 1.00, p = 0.05; purging OR: 0.63, 95%CI: 0.32 to 1.27, p = 0.20; fasting OR: 0.63, 95%CI: 0.38 to 1.07, p = 0.09). There was no evidence of interactions between either exposures and time.

**Table 3:** Multilevel logistic models of the association between tertiles of IL6 and CRP and disordered eating behaviors and diagnoses at age 14, 16, and 18. Samples of participants with complete exposure and at least one outcome measurements (n = 3,480, imputed confounders and outcomes in those with at least one available).

Outcomes	Exposure: thirds of IL-6		Exposure: thirds of CRP	
	2nd third (vs 1st) OR (95%CI) of outcomes	3rd third (vs 1st) OR (95%CI) of outcomes	2nd third (vs 1st) OR (95%CI) of outcomes	3rd third (vs 1st) OR (95%CI) of outcomes
**Fasting**				
*Univariable*	1.68 (1.16, 2.45) p = 0.006	1.97 (1.24, 3.14) p = 0.004	2.22 (1.49, 3.29) p = 0.001	1.95 (1.22, 3.13) p = 0.006
*Multivariable model1*	1.13 (0.77, 1.65) p = 0.53	1.06 (0.66, 1.71) p = 0.79	1.14 (0.77, 1.70) p = 0.52	0.65 (0.38, 1.10) p = 0.10
*Multivariable model2*	1.05 (0.72, 1.53) p = 0.81	0.98 (0.62, 1.57) p = 0.94	1.14 (0.77, 1.69) p = 0.51	0.63 (0.38, 1.07) p = 0.09
**Dieting**				
*Univariable*	1.66 (1.19, 2.31) p = 0.003	2.36 (1.60, 3.47) p < 0.0001	2.67 (1.89, 3.76) p < 0.0001	4.05 (1.89, 3.76) p < 0.0001
*Multivariable model1*	0.94 (0.68, 1.31) p = 0.73	0.91 (0.61, 1.35) p = 0.64	1.23 (0.87, 1.73) p = 0.23	0.96 (0.61, 1.50) p = 0.86
*Multivariable model2*	0.92 (0.66, 1.28) p = 0.63	0.89 (0.60, 1.32) p = 0.56	1.23 (0.88, 1.73), p = 0.23	0.95 (0.61, 1.48), p = 0.81
**Binge eating**				
*Univariable*	1.21 (0.87, 1.68) p = 0.25	1.30 (0.87, 1.92) p = 0.20	1.43 (1.01, 2.01), p = 0.04	1.39 (0.90, 2.26) p = 0.14
*Multivariable model1*	0.90 (0.65, 1.26) p = 0.56	0.83 (0.55, 1.25) p = 0.37	0.90 (0.64, 1.26) p = 0.54	0.61 (0.38, 1.00) p = 0.05
*Multivariable model2*	0.87 (0.63, 1.21) p = 0.42	0.80 (0.53, 1.20) p = 0.27	0.91 (0.65, 1.28) p = 0.59	0.62 (0.39, 1.00) p = 0.05
**Purging**				
*Univariable*	1.53 (0.90, 2.56) p = 0.11	1.77 (0.90, 3.47) p = 0.09	1.95 (1.16, 3.32) p = 0.01	1.91 (1.00, 3.62) p = 0.05
*Multivariable model1*	0.98 (0.58, 1.66) p = 0.94	0.94 (0.47, 1.85) p = 0.84	1.01 (0.60, 1.68) p = 0.98	0.63 (0.32, 1.26), p = 0.19
*Multivariable model2*	0.95 (0.56, 1.60) p = 0.84	0.90 (0.46, 1.76) p = 0.76	1.03 (0.62, 1.70), p = 0.92	0.63 (0.32, 1.27), p = 0.20

	Exposure: thirds of IL-6			
	2nd third (vs 1st) OR (95%CI) of outcomes	3rd third (vs 1st) OR (95%CI) of outcomes	2nd third (vs 1st) OR (95%CI) of outcomes	3rd third (vs 1st) OR (95%CI) of outcomes

**Any disordered eating**				
*Univariable*	1.58 (1.23, 2.01), p<0.0001	2.07 (1.55, 2.76),p<0.0001	1.88 (1.46, 2.41), p<0.0001	3.31 (1.72, 3.13), p<0.0001
*Multivariable model1*	1.02 (0.80, 1.31), p=0.84	1.05 (0.80, 1.40), p=0.72	0.98 (0.76, 1.25), p=0.84	0.71 (0.51, 0.98), p=0.03
*Multivariable model2*	0.99 (0.77, 1.25), p=0.91	1.00 (0.78, 1.25), p=0.97	0.98 (0.77, 1.25), p=0.87	0.70 (0.51, 0.97), p=0.03
**Anorexia Nervosa**				
*Univariable*	1.59 (0.60, 4.17) p=0.34	1.56 (0.52, 4.63) p=0.43	2.05 (0.85, 4.92) p=0.11	0.88 (0.25, 3.10) p=0.85
*Multivariable model1*	1.35 (0.51, 3.64) p=0.54	1.28 (0.41, 3.96) p=0.67	1.47 (0.59, 3.59) p=0.41	0.65 (0.17, 2.44) p=0.52
*Multivariable model2*	1.30 (0.49, 3.45) p=0.59	1.20 (0.39, 3.71) p=0.75	1.51 (0.62, 3.70) p=0.36	0.65 (0.18, 2.41) p=0.52
**Bulimia Nervosa**				
*Univariable*	1.21 (0.64, 2.28) p=0.55	1.32 (0.62, 2.81) p=0.47	2.50 (1.27, 4.96) p=0.009	1.36 (0.54, 3.47) p=0.52
*Multivariable model1*	0.88 (0.43, 1.65), p=0.68	0.80 (0.37, 1.77), p=0.59	1.56 (0.22, 1.59) p=0.28	0.57 (0.21, 1.59) p=0.28
*Multivariable model2*	0.84 (0.45, 1.59) p=0.59	0.76 (0.34, 1.67) p=0.49	1.54 (0.76, 3.09) p=0.23	0.55 (0.20, 1.55) p=0.25
**Binge eating disorder**				
*Univariable*	1.39 (0.68, 2.87) p=0.37	1.55 (0.66, 3.61) p=0.31	1.41 (0.70, 2.87) p=0.33	1.61 (0.66, 3.96) p=0.30
*Multivariable model1*	0.97 (0.47, 2.00) p=0.93	0.83 (0.34, 2.02) p=0.68	0.80 (0.39, 1.64), p=0.55	0.60 (0.22, 1.61) p=0.31
*Multivariable model2*	0.92 (0.44, 1.90) p=0.81	0.76 (0.31, 1.85) p=0.54	0.82 (0.40, 1.67) p=0.58	0.60 (0.22, 1.61) p=0.31

**Multivariable model 1**: adjusted for sex, time, and total fat mass in grams at age 9 years. **Multivariable model 2:** adjusted for sex, time, total fat mass in grams at age 9 years, maternal education, maternal age at child’s birth, maternal pre-pregnancy total fat mass in grams, maternal depression in pregnancy, mental health difficulties (i.e., total strength and difficulties questionnaire score) at age 7 years, peer victimization at age 8 years, maternal experience of stressful life events in pregnancy.

### 3.4 Associations between IL-6 and CRP at age nine and eating disorders during adolescence

We observed a similar pattern of results using eating disorder diagnoses as outcomes (Table 3). In univariable models, children in the middle and top third of IL-6 and CRP had greater odds of all diagnoses in adolescence, with the exception of anorexia nervosa in relation to CRP. Nevertheless, for all these associations, 95% confidence intervals were wide and included the null. After adjustment for sex and fat mass in model one, most of these associations were reduced (particularly those pertaining to the top third of IL-6 and CRP) and remained unaltered in model two. Here, we found that children in the top third of CRP had lower odds of all diagnoses (anorexia nervosa OR: 0.65, 95%CI: 0.18 to 2.41, p = 0.52; bulimia nervosa OR: 0.55, 95%CI: 0.20, 1.55, p = 0.25, binge-eating disorder OR: 0.60, 95%CI: 0.22 to 1.61, p = 0.31) even though 95% CIs were wide and p-values large. Children in the top third of IL-6 also had lower odds of both bulimia nervosa (OR: 0.76, 95%CI: 0.34 to 1.67, p = 0.49) and binge eating disorder (OR: 0.76, 95%CI: 0.31 to 1.85, p = 0.54). On the contrary, the odds of anorexia nervosa for those in the middle and top third of IL-6 were not reduced as much after adjustment for confounders (middle third, model two OR: 1.30, 95%CI: 0.49 to 3.45, p = 0.59; top third model two OR: 1.20, 95%CI: 0.39 to 3.71, p = 0.75), although there was no evidence of an association. There was also no evidence of interactions between any of the exposures and time.

### 3.5 Associations between IL-6 and CRP at age nine and eating disorder cognitions at age 14

In univariable models (Table 4) children in the middle and top third of IL-6 and CRP had greater levels of weight and shape concerns and body dissatisfaction. These associations disappeared after adjustment for sex and fat mass in model one and remained unaltered in the fully adjusted model two.

**Table 4:** Linear regression models for the association between tertiles of IL-6 and CRP and body dissatisfaction and weight and shape concerns at age 14 years (n = 2,901).

Outcomes	Exposure: third of IL-6		Exposure: third of CRP	
	2nd third (vs 1st) Mean difference (95%CI) in outcome	3rd third (vs 1st) Mean difference (95%CI) in outcome	2nd third (vs 1st) Mean difference (95%CI) in outcome	3rd third (vs 1st) Mean difference (95%CI) in outcome
**Body dissatisfaction**				
*Univariable*	1.66 (1.00, 2.32) p < 0.0001	2.48 (1.68, 3.30) p < 0.0001	2.60 (1.96, 3.24) p < 0.0001	4.11 (3.28, 4.92) p < 0.0001
*Multivariable model1*	0.01 (-0.59, 0.62) p = 0.96	0.06 (-0.70, 0.82) p = 0.88	0.54 (-0.07, 1.16) p = 0.08	0.22 (-0.61, 1.05) p = 0.60
*Multivariable model2*	−0.06 (-0.66, 0.55) p = 0.61	0.01 (-0.75, 0.76) p = 0.98	0.55 (-0.07, 1.17) p = 0.08	0.22 (-0.61, 1.05) p = 0.60
**Weight & shape concerns**				
*Univariable*	0.41 (0.25, 0.57) p < 0.0001	0.65 (0.46, 0.84), p < 0.0001	0.62 (0.47, 0.78) p < 0.0001	0.91 (0.72, 1.10) p < 0.0001
*Multivariable model1*	0.03 (-0.11, 0.17) p = 0.64	0.09 (-0.08, 0.27), p = 0.28	0.14 (-0.01, 0.29), p = 0.05	0.03 (-0.16, 0.22) p = 0.76
*Multivariable model2*	0.01 (-0.13, 0.15), p = 0.84	0.08 (-0.10, 0.25) p = 0.38	0.15 (0.01, 0.29) p = 0.05	0.03 (-0.16, 0.22) p = 0.75

**Multivariable model 1**: adjusted for sex and total fat mass at age 9 years. **Multivariable model 2**: adjusted for sex, total fat mass at age 9 years, maternal education, maternal age at child’s birth, maternal pre-pregnancy BMI, maternal depression in pregnancy, mental health difficulties (i.e., total strength and difficulties questionnaire score) at age 7 years, peer victimization at age 8 years, maternal experience of stressful life events in pregnancy.

### 3.6 Sensitivity analyses using IL-6 and CRP as continuous measures

Broadly, these results were similar to those of the main analyses (eTables 3,4). We did not observe any associations between IL6 and any of the outcomes. However, in multivariable model two, a standard deviation increase in CRP was associated with lower odds of any disordered eating (OR: 0.87, 95%CI: 0.77 to 0.99, p = 0.03), fasting (OR: 0.79, 95%CI: 0.64 to 0.96, p = 0.02), and purging (OR: 0.73, 95%CI: 0.55 to 0.99, p = 0.04), albeit weakly so. There was also some evidence of a non-linear association between CRP (CRP quadratic term p-value = 0.09) and lower odds of binge eating (OR: 0.74, 95%CI: 0.55 to 0.99, p = 0.04). There were comparable associations between higher CRP and lower odds of anorexia nervosa (OR: 0.53, 95%CI: 0.24 to 1.22, p = 0.14) and bulimia nervosa (OR: 0.71, 95%CI: 0.43 to 1.17, p = 0.18).

In analyses run on complete cases (eTables 5,6), the only association we observed was for lower odds of binge eating for children in the top third of CRP (OR: 0.57, 95%CI: 0.35, 0.95, p = 0.03; eTable 5). All other estimates were nevertheless compatible with those observed in imputed data.

## 4 Discussion

In this study, we found no evidence of an association between serum levels of IL-6 at age nine years and all of the outcomes investigated measured in adolescence. There was weak evidence of an association between high levels of CRP at age nine years and lower odds of fasting, binge eating, and purging at ages 14 to 18 years. Although we adjusted our analyses for sex, fat mass, pre-existing psychopathology, bullying, and socio-demographic indicators, we cannot exclude the potential of a chance finding or residual confounding, particularly in light of: the weak associations found; the number of tests conducted; the observation that adjusting for confounding led to a large reduction in the associations; and the fact that we could not control for a number of potential confounders identified in our DAG. Low statistical power could also explain the weak associations we observed since the prevalence of our outcomes was low and the confidence intervals around our estimates wide.

### 4.1 Comparison with previous literature

Previous studies reported that women with anorexia nervosa have higher levels of IL-6, but that these returned within the normal range after weight restoration and improvement of psychological symptoms (Dalton et al., 2018a, Solmi et al., 2015). This suggests that immune alterations could be a consequence of illness rather than be its cause. Likewise, we did not observe any longitudinal associations between IL-6, restrictive eating behaviours, weight and shape concerns, body dissatisfaction (common phenotypical presentations of anorexia nervosa), or anorexia nervosa. Therefore, we propose that, taken together, existing evidence suggests that IL-6 alterations could be a state marker of anorexia nervosa rather than a risk factor for its development.

Case-control studies suggest that women with anorexia nervosa may have lower levels of CRP (Solmi et al., 2015), although no studies have investigated whether this association remains after weight restoration. Despite differences in design and populations, our findings broadly align with those of previous studies. Specifically, we found that higher levels of CRP were associated with lower odds of fasting with similar patterns observed when using anorexia nervosa as outcome (although, here, statistical evidence of an association was absent). We also found similar associations between higher levels of CRP and lower odds of binge eating, purging, and bulimia nervosa (although for the latter two evidence of an association was weak and absent, respectively). This is in contrast with previous evidence of higher levels of CRP in people with loss of control eating (Shank et al., 2016, Succurro et al., 2015). However, it is possible that metabolic sequalae of binge eating, e.g. dyslipidaemia (Hudson et al., 2010, Tanofsky-Kraff et al., 2012, Yoon et al., 2019), could lead to higher levels of CRP, whereas our findings could reflect aetiological mechanisms.

### 4.2 Strengths and limitations of this study

Strengths of this study include a prospective design, the use of a large sample, and adjustment for multiple relevant confounders. Limitations include limited inflammatory marker data. We only had data on IL-6 and CRP at one time point in childhood. Future studies should incorporate a wider range of markers at several time points in childhood, as inflammation is a dynamic process and understanding ‘at risk’ developmental stages would aid aetiological inferences. Disordered eating behaviours were self-reported, which could have resulted in misclassification, as eating disorders can be highly ego-syntonic. However, the questions we included have been previously used in a number of studies in ALSPAC (Bornioli et al., 2019, Reed et al., 2017, Solmi et al., 2019) and other samples (Chin et al., 2018, Field et al., 2012, Haines et al., 2009) and produced consistent results suggesting that they likely capture core eating disorder presentations. The prevalence of eating disorder diagnoses, as expected, was low, though in line with their known epidemiology (Smink et al., 2013). This likely resulted in low statistical power and type II errors. We nevertheless included these outcomes to explore the general pattern of association between the exposures and more severe outcome presentations. We cannot exclude the potential for residual confounding, including genetic confounding. Although there is a large GWAS of anorexia nervosa (Watson et al., 2019), currently there are no adequately powered GWAS of disordered eating behaviors and related cognitions (Boraska et al., 2012, Wade et al., 2013), bulimia nervosa, or binge-eating disorder. Finally, a large proportion of the participants were lost to follow up. To address this limitation, we imputed missing confounder data under a missing at random assumption and compared estimates with complete cases. We were reassured by the fact that results were largely comparable, especially for the final models that included the main drivers of missingness.

### 4.3 Interpretation of findings

The overall weak evidence we observe calls for caution when interpreting our results. One biologically plausible explanation for our findings is that – whilst cross-sectionally observed low levels of CRP in anorexia nervosa could reflect immunosuppression following severe weight loss – premorbid high levels of CRP could have anorexigenic effects, and appetitive traits have been shown to be associated with eating disorders (Herle et al., 2019). In both humans and mice, naturally-occurring or experimentally-induced inflammation results in ‘sickness’ behaviour, characterised by low mood, reduced appetite and weight loss (Dantzer, 2009). High levels of CRP (and IL-6) are believed to be the main cause of cachexia—a syndrome whose symptoms include anorexia and early satiety (Mahmoud and Rivera, 2002, Morley et al., 2006)—and anorexia in elderly populations (Morley, 2001). It is possible that greater inflammation in childhood could act as an appetite-suppressing factor. This could confer some degree of protection against binge eating, by reducing overeating, a precursor of binge eating behaviours in adolescence (Herle et al., 2019). The association with lower odds of restricting behaviours (i.e., fasting and purging) might be reflect fewer compensating behaviours in the absence of binge eating. Appetitive traits (and, specifically, the effect that CRP exerts on them) could represent one of many risk factors with an overall small effect that our sample is unable to detect with sufficient precision. However, as this is the first study that has investigated these associations, and we only observed weak associations, replication of these findings in even larger samples is warranted before hypotheses around potential mechanisms are formalised. Meanwhile, the overall lack of strong evidence for an association between inflammatory markers and eating disorders appears to be in contrast with what has been previously observed for other conditions including as depression (Khandaker et al., 2019, Khandaker et al., 2018, Khandaker et al., 2014, Valkanova et al., 2013), often comorbid with eating disorders, suggesting different aetiological pathways.
