# Supplementary material for: Longitudinal associations between circulating interleukin-6 and C-reactive protein in childhood, and eating disorders and disordered eating in adolescence
Source: Brain Behav Immun. 2020 Oct;89:491–500. doi: 10.1016/j.bbi.2020.07.040 (PMC7902903; doi:10.1016/j.bbi.2020.07.040)
Supplement: Supplementary data 1 [file mmc1.docx]

**Longitudinal associations between circulating Interleukin-6 and C-Reactive Protein in childhood, and eating disorders and disordered eating in adolescence**

*Francesca Solmi PhD^1^, Cynthia M. Bulik PhD ^2, 3, 4^, Bianca L. De Stavola Ph ^5^, Christina Dalman PhD ^4^, Golam M. Khandaker PhD ^6,7^, Glyn Lewis PhD ^1^*

**^1^** Division of Psychiatry, UCL, London, UK.

^2^ Department of Psychiatry, University of North Carolina at Chapel Hill, Chapel Hill, USA

^3^ Department of Nutrition, University of North Carolina at Chapel Hill, Chapel Hill, USA

^4^ Department of Medical Epidemiology and Biostatistics, Karolinska Institutet, Stockholm, Sweden

^5^ Great Ormond Street Institute of Child Health, UCL, London UK

^6^ University of Cambridge, Cambridge, UK

*^7^* Cambridgeshire and Peterborough NHS Foundation Trust, Cambridge, UK

**Corresponding author**

Francesca Solmi, PhD

UCL Division of Psychiatry

Maple House, 6^th^ Floor, Wing A

149 Tottenham court road,

W1T 7NF, London

Email: [Francesca.solmi@ucl.ac.uk](mailto:Francesca.solmi@ucl.ac.uk)

**Table of contents**

[eMethods1: eating disorder measures 3](#_Toc45547761)

[eMethods 2: confounders 6](#_Toc45547762)

[eFigure 2: Distribution of standardized IL6 values in the sample of children with complete exposure data 7](#_Toc45547763)

[eFigure 3: Distribution of standardized CRP values in the sample of children with complete exposure data 7](#_Toc45547764)

[eMethods 3: Multiple imputation 8](#_Toc45547765)

[eTable1: Predictors of missing data at age 9 assessment, missing only blood sample data at age 9 assessment vs. having data available at age 9 on all measures. (having data available - n=4,652 - is the reference outcome category) 9](#_Toc45547766)

[eTable 2: Predictors of missing outcome data by sample definition among ALSPAC participants with complete exposure data. 10](#_Toc45547767)

[eTable 3: Multilevel logistic models of the association between continuous measurements IL6 and CRP and disordered eating behaviors and diagnoses at age 14, 16, and 18. Samples of participants with complete exposure and at least one outcome measurements (n=3,480, imputed confounders and outcomes in those with at least one available). 11](#_Toc45547768)

[eTable 4: Linear regression models for the association between continuous measurements of IL6 and CRP and body dissatisfaction and weight and shape concerns at age 14 years (n= 2,673) 13](#_Toc45547769)

[eTable 5: Multilevel logistic models of the association between thirds of IL6 and CRP and disordered eating behaviors and diagnoses at age 14, 16, and 18. Samples of participants with complete exposure and at least one outcome measurements (n=2,040, complete cases). 14](#_Toc45547770)

[eTable 6: Linear regression models for the association between tertiles of IL6 and CRP and body dissatisfaction and weight and shape concerns at age 14 years (n= 1,751, complete cases) 16](#_Toc45547771)

[References 17](#_Toc45547772)

## eMethods1: eating disorder measures

##

Disordered eating measures

Adolescents were asked how often in the previous 12 months had fasted (i.e., not eating for 24 hours) or purged (i.e., self-induced vomiting, or used of laxatives/diet pills) for weight loss. We defined these behaviors as present if the adolescents reported them to occur at least monthly and up to daily. We defined binge eating using a two-part question. First, adolescents were asked how often they had eaten a large amount of food in short period of time. Those who reported the behaviors as occurring (regardless of frequency) were asked to report whether during these episodes they felt out of control (possible answers: “never/sometimes/usually”). We considered adolescents as having experienced binge eating if they reported episodes of overeating occurring monthly (and up to daily) and ‘sometimes’ or ‘usually’ accompanied by loss of control. Participants were also asked if they had dieted in the previous year and, if so, for how long. We consider adolescents as dieting if they reported dieting ‘several times’, ‘often’, or being ‘always’ on a diet and said that they dieted for at least one month and up to 12 months continuously. These definitions have previously been used in this sample.

Eating disorder diagnoses, were defined as follows:

**Anorexia nervosa**. DSM5 criteria for anorexia nervosa are:

1. Restriction of energy intake relative to requirements, leading to a significantly low body weight in the context of age, sex, developmental trajectory, and physical health.
2. Intense fear of gaining weight or of becoming fat, or persistent behaviour that interferes with weight gain, even though at a significantly low weight.
3. Disturbance in the way in which one’s body weight or shape is experienced, undue influence of body weight or shape on self-evaluation, or persistent lack of recognition of the seriousness of the current low body weight

As measures of body dissatisfaction were only available at age 14 years, we limited our definition of anorexia nervosa to its behavioural and BMI components.

We defined a participant as having anorexia nervosa if they had:

1. weekly episodes of fasting for weight loss AND dieting – criterion 1 and 2
2. An underweight or normal BMI – criterion 1 and 2

**Bulimia Nervosa.** DSM5 criteria for bulimia nervosa are:

1. Recurrent episodes of binge eating. An episode of binge eating is characterized by both:
   1. Eating in a discrete period of time (e.g. within any 2 hour period), an amount of food that is definitely larger than what most individuals would eat in a similar period of time under similar circumstances;
   2. A sense of lack of control over eating during the episodes (e.g. a feeling that one cannot stop eating or control what or how much one is eating.
2. Recurrent inappropriate compensatory behaviors to prevent weight gain, such as self-induced vomiting; misuse of laxatives, diuretics, or other medications; fasting; or excessive exercise.
3. The binge eating and inappropriate compensatory behaviors both occur, on average, at least once a week for 3 months.
4. Self-evaluation is unduly influenced by body shape and weight.
5. The disturbance does not occur exclusively during episodes of anorexia nervosa.

As measures of body dissatisfaction were only available at age 14 years, we limited our definition of bulimia nervosa to criteria 1, 2, 3, and 5.

We defined a participant as having bulimia nervosa if they had:

1. weekly episodes of binge eating (defined as explained above in ‘disordered eating measures’) – criteria 1 and 3
2. followed by compensatory behaviors (purging or fasting) indicating purging or non-purging bulimia nervosa. – criteria 2 and 4.
3. If an adolescent reported these behaviors and had an underweight BMI was coded has having anorexia nervosa (i.e., of the binge-purge sub-type) – criteria 5.

**Binge-eating disorder.** DSM5 criteria for binge eating disorder are:

1. Recurrent episodes of binge eating. An episode of binge eating is characterized by both:
   1. Eating in a discrete period of time (e.g. within any 2 hour period), an amount of food that is definitely larger than what most individuals would eat in a similar period of time under similar circumstances;
   2. A sense of lack of control over eating during the episodes (e.g. a feeling that one cannot stop eating or control what or how much one is eating).
2. Binge eating episodes are associated with three or more of the following:
   1. Eating much more rapidly than normal.
   2. Eating until feeling uncomfortably full.
   3. Eating large amounts of food when not feeling physically hungry.
   4. Eating alone because of feeling embarrassed by how much one is eating.
   5. Feeling disgusted with oneself, depressed, or very guilty afterwards.
3. Marked distress regarding binge eating is present.
4. The binge eating occurs, on average, at least once a week for 3 months.
5. The binge eating is not associated with the recurrent use of inappropriate compensatory behavior as in bulimia nervosa and does not occur exclusively during the course of bulimia nervosa or anorexia nervosa.

We defined a participant as having binge eating disorder if they had:

1. Weekly episodes of binge eating – criteria 1 and 4.
2. Absence of compensatory behaviours – criterion 5
3. At least three of the following criteria present: participant ate when not hungry, ate until uncomfortably full, ate faster, ate alone, felt guilty about over-eating – criterion 2

## eMethods 2: confounders

Confounders included in analyses

At nine years of age, children attended a clinic appointment where their total fat mass (in grams) was measured with a whole body DXA scan performed using a Lunar prodigy narrow fan beamer densitometer. Pre-existing mental health problems were parentally-reported using the Strength and Difficulties Questionnaire (SDQ), a 40-item questionnaire measuring internalizing (peer and emotional) and externalizing (hyperactivity and conduct) problems.(3) We used the total scale score as a continuous variable. At a clinical assessment conducted at age eight years, children were also interviewed about peer victimization using a modified version of the Bullying and Friendship Interview Schedule.(4)

Maternal age at child’s birth was calculated by the ALSPAC study team by subtracting the date of delivery from the mother’s date of birth. We derived maternal pre-pregnancy BMI from values of height and pre-pregnancy weight self-reported at 18 weeks of gestation. At the same assessments, mothers answered a battery of 40 questions related to stressful events (ranging from health, legal, and financial issues to pregnancy-related worries and bereavement), which might have occurred in the early pregnancy period. Each question was scored on a five-point Likert scale ranging from ‘did not occur’ to ‘occurred and was severely affected by it’.(5) Answers from all questions were added to obtain a total score, where higher values indicated greater number and impact of stressful events occurred.

Mothers also reported their depressive symptoms at 32 weeks of gestation using the Edinburgh Postnatal Depression Scale, a 10-item scale measuring depressive symptoms in the pre-natal and post-natal period.(6)

## eFigure 2: Distribution of standardized IL6 values in the sample of children with complete exposure data

**
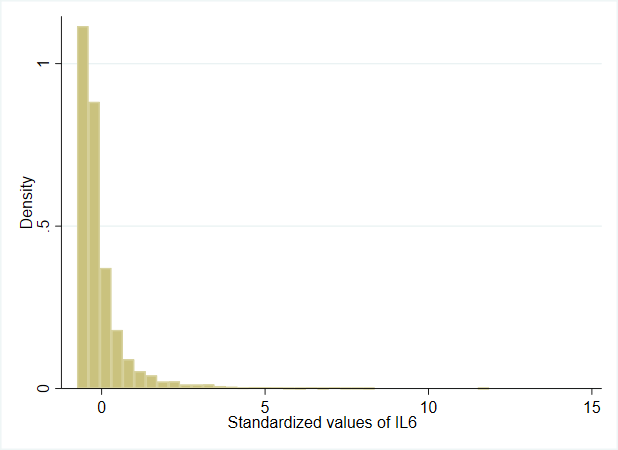
**

## eFigure 3: Distribution of standardized CRP values in the sample of children with complete exposure data

**
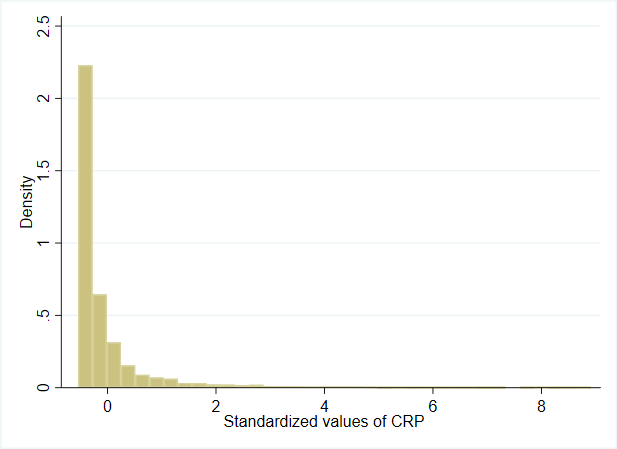
**

## eMethods 3: Multiple imputation

In our prediction models we included all variables used in the analytical models, plus a number of auxiliary variables, which we hypothesised or knew—based on previous research —to be associated both with the variables to be imputed and attrition in the sample. These were: ethnicity, age and sex-standardized z-scores for BMI at age 7, 9, 10, 11, 14, 16, and 18 years; depressive symptoms (total score of the Short Moods and Feelings Questionnaire(7)) at age 11, 14, 16, and 18 years; serum levels of leptin at age 9 years; total scores of the Strengths and Difficulties Questionnaire at age 9 years; child’s self-esteem measured at age 8 years; sexual orientation measured at 15 years; child’s total IQ measured at age 8 years; self-harm in previous 12 months at age 16 years; autistic social traits measured with the Social Communication Disorder Checklist(8) at age 7, 11, 14, and 16 years; emotional, restrained, and external eating measured at 14 years with a subset of items from the Dutch Eating Behaviour Questionnaire.(9)

## eTable1: Predictors of missing data at age 9 assessment, missing only blood sample data at age 9 assessment vs. having data available at age 9 on all measures. (having data available - n=4,652 - is the reference outcome category)

|  | **No data available at clinic assessment at age 9**  **N=6,582**  **RRR (95%CI)** | **Clinic data available, but no blood sample at age 9**  **N=2,553**  **RRR (95%CI)** |
| --- | --- | --- |
| **Sex** |  |  |
| *Male* | Reference | Reference |
| *Female* | 0.88 (0.81, 0.95) | 1.12 (1.02, 1.23) |
| **Child’s Mental health difficulties at age 7 years** |  |  |
| *Absent* | Reference | Reference |
| *Present* | 1.52 (1.18, 1.96) | 1.36 (1.05, 1.77) |
| **Maternal Education** |  |  |
| *Compulsory* | Reference | Reference |
| *Non-compulsory* | 0.44 (0.41, 0.49) | 0.82 (0.74, 0.91) |
| **Maternal age** |  |  |
| *15-19* | Reference | Reference |
| *20-25* | 0.39 (0.31, 0.50) | 0.85 (0.60, 1.22) |
| *26-35* | 0.17 (0.14, 0.22) | 0.79 (0.57, 1.13) |
| *36-44* | 0.15 (0.11, 0.20) | 0.81 (0.56, 1.20) |
| **Maternal pre-pregnancy BMI** |  |  |
| *Underweight* | Reference | Reference |
| *Normal weight* | 0.73 (0.60, 0.88) | 0.88 (0.69, 1.13) |
| *Overweight* | 0.79 (0.64, 0.98) | 0.96 (0.73, 1.26) |
| *Obese* | 0.89 (0.69, 1.15) | 1.12 (0.81, 1.54) |
| **Maternal depression** |  |  |
| *No* | Reference | Reference |
| *Yes* | 1.74 (1.54, 1.96) | 1.18 (1.01, 1.38) |
| **Peer victimization at 8 years** |  |  |
| *No* | Reference | Reference |
| *Yes* | 1.31 (1.11, 1.55) | 1.14 (1.02, 1.27) |
| **Thirds of Stressful Life Events score** |  |  |
| *1^st^ third (lowest)* | Reference | Reference |
| *2^nd^ third* | 0.89 (0.81, 0.98) | 0.98 (0.87, 1.11) |
| *3^rd^ third (highest)* | 1.22 (1.01, 1.35) | 1.16 (1.032 1.31) |

List of abbreviations: RRR= relative risk ratio

## eTable 2: Predictors of missing outcome data by sample definition among ALSPAC participants with complete exposure data.

|  | **No disordered eating measurements available in adolescence (14, 16, 18 years)**  **N=1,172 (25.2%)** | **At least one eating behavior or cognition missing at 14 years**  **1,751 (37.6%)** |
| --- | --- | --- |
| **IL6** | 1.00 (0.96, 1.05), p=0.81 | 1.00 (0.96, 1.05), p=0.78 |
| **CRP** | 0.96 (0.89, 1.02), p=0.20 | 0.99 (0.94, 1.05), p=0.81 |
| **Sex** |  |  |
| *Male* | Reference | Reference |
| *Female* | 0.43 (0.38, 0.50), p<0.0001 | 0.54 (0.48, 0.61), p<0.0001 |
| **Thirds of total fat mass** |  |  |
| *1^st^ third (lowest)* | Reference | Reference |
| *2^nd^ third* | 0.86 (0.73, 1.01), p=0.07 | 0.85 (0.73, 0.97), p=0.02 |
| *3^rd^ third (highest)* | 0.87 (0.74, 1.03), p=0.09 | 0.87 (0.75, 1.01), p=0.07 |
| **Child’s Mental health difficulties at age 7 years** |  |  |
| *Absent* | Reference | Reference |
| *Present* | 1.52 (1.04, 2.22), p=0.03 | 1.36 (0.96, 1.91), p=0.09 |
| **Maternal Education** |  |  |
| *Compulsory* | Reference | Reference |
| *Non-compulsory* | 0.46 (0.40, 0.53), p<0.0001 | 0.57 (0.51, 0.64) p<0.0001 |
| **Maternal age** |  |  |
| *15-19* | Reference | Reference |
| *20-25* | 0.65 (0.41, 1.02), p=0.06 | 0.57 (0.36, 0.90), p=0.01 |
| *26-35* | 0.37 (0.24, 0.58), p<0.0001 | 0.38 (0.24, 0.59), p<0.0001 |
| *36-44* | 0.27 (0.16, 0.45), p<0.0001 | 0.36 (0.22, 0.59), p<0.0001 |
| **Maternal pre-pregnancy BMI** |  |  |
| *Underweight* | Reference | Reference |
| *Normal weight* | 1.11 (0.77, 1.61), p=0.55 | 1.22 (0.89, 1.68), p=0.22 |
| *Overweight* | 1.67 (1.12, 2.47), p=0.01 | 1.56 (1.10, 2.21), p=0.01 |
| *Obese* | 1.54 (0.97, 2.45), p=0.07 | 1.55 (1.02, 2.34), p=0.04 |
| **Maternal depression** |  |  |
| *No* | Reference | Reference |
| *Yes* | 1.42 (1.15, 1.75), p=0.0013 | 1.35 (1.11, 1.65), p=0.003 |
| **Peer victimization at 8 years** |  |  |
| *No* | Reference | Reference |
| *Yes* | 1.28 (1.09, 1.49), p=0.0019 | 1.24 (1.08, 1.43) p=0.002 |
| **Thirds of Stressful Life Events score** |  |  |
| *1^st^ third (lowest)* | Reference | Reference |
| *2^nd^ third* | 1.05 (0.89, 1.24), p=0.54 | 1.03 (0.90, 1.20), p=0.61 |
| *3^rd^ third (highest)* | 1.11 (0.93, 1.32), p=0.25 | 1.17 (1.00, 1.37), p=0.05 |

## eTable 3: Multilevel logistic models of the association between continuous measurements IL6 and CRP and disordered eating behaviors and diagnoses at age 14, 16, and 18. Samples of participants with complete exposure and at least one outcome measurements (n=3,480, imputed confounders and outcomes in those with at least one available).

| Outcomes | **IL6** | | **CRP** | |
| --- | --- | --- | --- | --- |
|  | **IL6**  **OR (95%CI)** | **IL6^2^**  **OR (95%CI)** | **CRP**  **OR (95%CI)** | **CRP^2^**  **OR (95%CI)** |
| **Fasting** |  |  |  |  |
| *Univariable* | 1.22 (1.05, 1.43) p=0.01 | - | 1.05 (0.91, 1.22) p=0.51 |  |
| *Multivariable model1* | 1.07 (0.90, 1.27) p=0.46 | - | 0.81 (0.66, 0.98) p=0.03 |  |
| *Multivariable model2* | 1.03 (0.87, 1.26) p=0.73 | - | 0.79 (0.64, 0.96) p=0.02 |  |
| *Multivariable model2** | 1.09 (0.80, 1.49) p=0.57 | 0.98 (0.91, 1.06) p=0.64 | 0.74 (0.53, 1.03) p=0.08 | 1.01 (0.94, 1.09) p=0.73 |
| **Dieting** |  |  |  |  |
| *Univariable* | 1.19 (1.03, 1.37) p=0.02 | - | 1.21 (1.07, 1.39) p=0.004 |  |
| *Multivariable model1* | 0.95 (0.80, 1.12) p=0.53 | - | 0.87 (0.73, 1.05) p=0.14 |  |
| *Multivariable model2* | 0.94 (0.80, 1.11) p=0.45 | - | 0.86 (0.72, 1.03) p=0.10 |  |
| *Multivariable model2** | 0.97 (0.73, 1.27) p=0.81 | 0.99 (0.92, 1.06) p=0.77 | 0.85 (0.63, 1.14) p=0.27 | 1.00 (0.94, 1.06) p=0.94 |
| **Binge eating** |  |  |  |  |
| *Univariable* | 1.04 (0.88, 1.22) p=0.65 |  | 1.06 (0.91, 1.24) p=0.42 |  |
| *Multivariable model1* | 0.93 (0.77, 1.11) p=0.39 |  | 0.90 (0.75, 1.09) p=0.31 |  |
| *Multivariable model2* | 0.90 (0.75, 1.08) p=0.26 |  | 0.90 (0.75, 1.08) p=0.25 |  |
| *Multivariable model2** | 0.92 (0.68, 1.22) p=0.56 | 0.99 (0.91, 1.07) p=0.85 | 0.73 (0.55, 0.99) p=0.04 | 1.05 (0.99, 1.10) p=0.09 |
| **Purging** |  |  |  |  |
| *Univariable* | 1.17 (0.91, 1.49) p=0.22 |  | 1.01 (0.80, 1.26) p=0.95 |  |
| *Multivariable model1* | 1.01 (0.77, 1.32) p=0.95 |  | 0.77 (0.55, 1.05) p=0.09 |  |
| *Multivariable model2* | 0.98 (0.75, 1.29) p=0.89 |  | 0.74 (0.54, 1.03) p=0.07 |  |
| *Multivariable model2** | 1.10 (0.71, 1.68) p=0.68 | 0.96 (0.84, 1.10) p=0.56 | 0.68 (0.41, 1.13) p=0.14 | 1.01 (0.88, 1.16) p=0.83 |

eTable 3: (continued)

|  |  | **IL6** | **CRP** | |
| --- | --- | --- | --- | --- |
|  | **IL6**  **OR (95%CI)** | **IL6^2^**  **OR (95%CI)** | **CRP**  **OR (95%CI)** | **CRP^2^**  **OR (95%CI)** |
| **Any disordered eating** |  |  |  |  |
| *Univariable* | 1.19 (1.07, 1.33), p=0.001 |  | 1.14 (1.02, 1.26), p=0.013 |  |
| *Multivariable model1* | 1.02 (0.91, 1.14), p=0.76 |  | 0.89 (0.79, 1.00), p=0.06 |  |
| *Multivariable model2* | 0.99 (0.89, 1.11), p=0.92 |  | 0.87 (0.77, 0.99), p=0.03 |  |
| *Multivariable model2** | 1.06(0.88, 1.29), p=0.52 | 0.98 (0.93, 1.03), p=0.40 | 0.79 (0.65, 0.97), p=0.03 | 1.02 (0.8, 1.06), p=0.31 |
| **Anorexia Nervosa** |  |  |  |  |
| *Univariable* | 1.10 (0.74, 1.60) p=0.66 |  | 0.67 (0.34, 1.31) p=0.24 |  |
| *Multivariable model1* | 1.03 (0.68, 1.54) p=0.92 |  | 0.55 (0.23, 1.28) p=0.17 |  |
| *Multivariable model2* | 0.99 (0.66, 1.51) p=0.98 |  | 0.53 (0.24, 1.22) p=0.14 |  |
| *Multivariable model2** | 1.41 (0.61, 3.23) p=0.42 | 0.86 (0.58, 1.26) p=0.43 | 1.01 (0.27, 3.77) p=0.98 | 0.41 (0.06, 2.95), p=0.37 |
| **Bulimia Nervosa** |  |  |  |  |
| *Univariable* | 1.02 (0.76, 1.29) p=0.88 |  | 0.96 (0.70, 1.32) p=0.81 |  |
| *Multivariable model1* | 0.89 (0.62, 1.28) p=0.54 |  | 0.75 (0.45, 1.20) p=0.22 |  |
| *Multivariable model2* | 0.85 (0.58, 1.24) p=0.39 |  | 0.71 (0.43, 1.17) p=0.18 |  |
| *Multivariable model2** | 0.98 (0.51, 1.90) p=0.96 | 0.92 (0.69, 1.23) p=0.59 | 0.55 (0.27, 1.14) p=0.11 | 1.05 (0.90, 1.24) p=0.55 |
| **Binge eating disorder** | |  |  |  |
| *Univariable* | 1.18 (0.90, 1.56) p=0.23 |  | 1.19 (0.94, 1.51), p=0.15 |  |
| *Multivariable model1* | 1.05 (0.76, 1.43) p=0.78 |  | 1.01 (0.76, 1.35) p=0.94 |  |
| *Multivariable model2* | 1.02 (0.74, 1.40) p=0.90 |  | 1.00 (0.79, 1.34) p=0.98 |  |
| *Multivariable model2** | 0.86 (0.49, 1.51) p=0.60 | 1.04 (0.93, 1.16) p=0.45 | 0.68 (0.41, 1.17), p=0.17 | 1.06 (0.98, 1.15), p=0.17 |

**Multivariable model 1**: adjusted for sex, time, and total fat mass in grams at age 9 years. **Multivariable model 2:** adjusted for sex, time, total fat mass in grams at age 9 years, maternal education, maternal age at child’s birth, maternal pre-pregnancy total fat mass in grams, maternal depression in pregnancy, mental health difficulties (i.e., total strength and difficulties questionnaire score) at age 7 years, peer victimization at age 8 years, maternal experience of stressful life events in pregnancy. **Multivariable model 2*:** multivariable model 2 + quadratic exposure term

## eTable 4: Linear regression models for the association between continuous measurements of IL6 and CRP and body dissatisfaction and weight and shape concerns at age 14 years (n= 2,673)

| **Outcomes** | **IL6** | | **CRP** | |
| --- | --- | --- | --- | --- |
|  | **IL6**  **Mean difference (95%CI)** | **IL6^2^**  **Mean difference (95%CI)** | **CRP**  **Mean difference (95%CI)** | **CRP^2^**  **Mean difference (95%CI)** |
| **Body dissatisfaction** |  |  |  |  |
| *Univariable* | 0.72 (0.40, 1.05) p<0.0001 |  | 0.96 (0.67, 1.25) p<0.0001 |  |
| *Multivariable model1* | 0.13 (-0.16, 0.43) p=0.38 |  | 0.06 (-0.22, 0.33) p=0.67 |  |
| *Multivariable model2* | 0.11 (-0.19, 0.41) p=0.47 |  | 0.03 (-0.24, 0.30) p=0.82 |  |
| *Multivariable model2** | 0.26 (-0.26, 0.77) p=0.33 | -0.04 (-0.16, 0.08) p=0.48 | -0.03 (-0.59, 0.52) p=0.92 | 0.01 (-0.09, 0.12) p=0.80 |
| **Weight & shape concerns** |  |  |  |  |
| *Univariable* | 0.18 (0.10, 0.25) p<0.0001 |  | 0.16 (0.10, 0.23) p<0.0001 |  |
| *Multivariable model1* | 0.05 (-0.03, 0.11) p=0.25 |  | -0.04 (-0.11, 0.02) p=0.18 |  |
| *Multivariable model2* | 0.03 (-0.03, 0.10) p=0.34 |  | -0.05 (-0.11, 0.01) p=0.11 |  |
| *Multivariable model2** | 0.07 (-0.05, 0.19) p=0.23 | -0.01 (-0.04, 0.02) p=0.41 | -0.05 (-0.18, 0.07) p=0.41 | 0.01 (-0.02, 0.02) p=0.95 |

**Multivariable model 1**: adjusted for sex and total fat mass at age 9 years

**Multivariable model 2**: adjusted for sex, total fat mass at age 9 years, maternal education, maternal age at child’s birth, maternal pre-pregnancy BMI, maternal depression in pregnancy, mental health difficulties (i.e., total strength and difficulties questionnaire score) at age 7 years, peer victimization at age 8 years, maternal experience of stressful life events in pregnancy. **Multivariable model 2*:** multivariable model 2 + quadratic exposure term

## eTable 5: Multilevel logistic models of the association between thirds of IL6 and CRP and disordered eating behaviors and diagnoses at age 14, 16, and 18. Samples of participants with complete exposure and at least one outcome measurements (n=2,040, complete cases).

| Outcomes | **Exposure: thirds of IL-6** | | **Exposure: thirds of CRP** | |
| --- | --- | --- | --- | --- |
|  | **2^nd^ third (vs 1^st^)**  **OR (95%CI) of outcomes** | **3^rd^ third (vs 1^st^)**  **OR (95%CI) of outcomes** | **2^nd^ third (vs 1^st^)**  **OR (95%CI) of outcomes** | **3^rd^ third (vs 1^st^)**  **OR (95%CI) of outcomes** |
| **Fasting** |  |  |  |  |
| *Univariable* | 1.28 (0.71, 2.30), p=0.41 | 1.45 (0.81, 2.61), p=0.21 | 2.48 (1.32, 4.68), p=0.005 | 2.49 (1.33, 4.65), p=0.005 |
| *Multivariable model1* | 1.04 (0.56, 1.91), p=0.91 | 0.88 (0.47, 1.64), p=0.68 | 1.52 (0.81, 2.87), p=0.20 | 1.04 (0.53, 2.06), p=0.91 |
| *Multivariable model2* | 0.92 (0.50, 1.69), p=0.79 | 0.75 (0.41, 1.39), p=0.36 | 1.59 (0.84, 3.00), p=0.15 | 1.11 (0.56, 2.17), p=0.77 |
| **Dieting** |  |  |  |  |
| *Univariable* | 1.63 (1.01, 2.64), p=0.05 | 3.14 (1.98, 2.64), p<0.0001 | 1.88 (1.14, 3.09), p=0.01 | 4.06 (2.53, 6.50), p<0.0001 |
| *Multivariable model1* | 1.19 (0.74, 1.91), p=0.48 | 1.43 (0.89, 2.29), p=0.14 | 1.05 (0.64, 1.73), p=0.84 | 1.30 (0.79, 2.13), p=0.30 |
| *Multivariable model2* | 1.17 (0.73, 1.87), p=0.52 | 1.35 (0.84, 2.16), p=0.21 | 1.08 (0.65, 1.76), p=0.77 | 1.31 (0.80, 2.15), p=0.28 |
| **Binge eating** |  |  |  |  |
| *Univariable* | 0.93 (0.58, 1.47), p=0.74 | 1.07 (0.68, 1.69), p=0.77 | 1.58 (1.02, 2.47), p=0.04 | 0.95 (0.59, 1.52), p=0.82 |
| *Multivariable model1* | 0.81 (0.51, 1.29) p=0.38 | 0.81 (0.51, 1.29), p=0.38 | 1.15 (0.74, 1.80), p=0.52 | 0.55 (0.33, 0.93), p=0.02 |
| *Multivariable model2* | 0.81 (0.52, 1.27), p=0.35 | 0.77 (0.48, 1.22), p=0.26 | 1.21 (0.78, 1.87), p=0.39 | 0.57 (0.35, 0.95), p=0.03 |
| **Purging** |  |  |  |  |
| *Univariable* | 1.89 (0.74, 4.89), p=0.18 | 1.50 (0.57, 3.91), p=0.41 | 2.20 (0.83, 5.87), p=0.11 | 2.44 (0.93, 6.38), p=0.07 |
| *Multivariable model1* | 1.42 (0.60, 3.34), p=0.42 | 0.88 (0.35, 2.20), p=0.78 | 1.32 (0.54, 3.22), p=0.54 | 1.04 (0.40, 2.66), p=0.94 |
| *Multivariable model2* | 1.41 (0.59, 3.35), p=0.44 | 0.83 (0.33, 2.10), p=0.70 | 1.42 (0.57, 3.50), p=0.45 | 1.10 (0.42, 2.83), p=0.85 |

|  | **Exposure: thirds of IL-6** | | **Exposure: thirds of CRP** | |
| --- | --- | --- | --- | --- |
|  | **2^nd^ third (vs 1^st^)**  **OR (95%CI) of outcomes** | **3^rd^ third (vs 1^st^)**  **OR (95%CI) of outcomes** | **2^nd^ third (vs 1^st^)**  **OR (95%CI) of outcomes** | **3^rd^ third (vs 1^st^)**  **OR (95%CI) of outcomes** |
| **Anorexia Nervosa** |  |  |  |  |
| *Univariable* | 1.68 (0.48, 5.87), p=0.41 | 1.12 (0.30, 4.26), p=0.87 | 2.79 (0.74, 10.56), p=0.12 | 1.13 (0.26, 4.88), p=0.86 |
| *Multivariable model1* | 1.71 (0.48, 6.08), p=0.41 | 1.08 (0.27, 4.26), p=0.91 | 2.12 (0.55, 7.96), p=0.27 | 0.95 (0.20, 4.46), p=0.95 |
| *Multivariable model2* | 1.53 (0.47, 5.01), p=0.48 | 0.99 (0.27, 3.64), p=0.99 | 2.51 (0.71, 8.90), p=0.15 | 1.09 (0.25, 4.80), p=0.90 |
| **Bulimia Nervosa** |  |  |  |  |
| *Univariable* | 0.68 (0.27, 1.75), p=0.43 | 0.94 (0.39, 2.25), p=0.89 | 2.74 (1.14, 6.61), p=0.02 | 0.92 (0.32, 2.64), p=0.87 |
| *Multivariable model1* | 0.66 (0.25, 1.69), p=0.38 | 0.85 (0.34, 2.11), p=0.73 | 2.38 (0.98, 5.82), p=0.06 | 0.76 (0.25, 2.36), p=0.63 |
| *Multivariable model2* | 1.53 (0.47, 5.01), p=0.48 | 0.99 (0.27, 3.63), p=0.99 | 2.51 (1.03, 6.15), p=0.04 | 0.78 (0.25, 2.43), p=0.68 |
| **Binge eating disorder** |  |  |  |  |
| *Univariable* | 1.15 (0.31, 4.33), p=0.84 | 1.58 (0.44, 5.68), p=0.49 | 2.92 (0.73, 11.80), p=0.13 | 2.53 (0.63, 10.31), p=0.19 |
| *Multivariable model1* | 0.91 (0.25, 3.36), p=0.89 | 0.86 (0.23, 3.25), p=0.83 | 1.87 (0.47, 7.43), p=0.38 | 1.09 (0.24, 4.79), p=0.92 |
| *Multivariable model2* | 0.91 (0.26, 3.21), p=0.89 | 0.81 (0.22, 2.97), p=0.76 | 2.00 (0.52, 7.66), p=0.31 | 1.13 (0.27, 4.73), p=0.86 |

eTable 5: (continued)

**Multivariable model 1**: adjusted for sex, time, and total fat mass in grams at age 9 years. **Multivariable model 2:** adjusted for sex, time, total fat mass in grams at age 9 years, maternal education, maternal age at child’s birth, maternal pre-pregnancy total fat mass in grams, maternal depression in pregnancy, mental health difficulties (i.e., total strength and difficulties questionnaire score) at age 7 years, peer victimization at age 8 years, maternal experience of stressful life events in pregnancy. **Multivariable model 3:** adjusted for all confounders included in model 2, plus fits an interaction between exposure and time, testing the presence of time-specific effects.

## eTable 6: Linear regression models for the association between tertiles of IL6 and CRP and body dissatisfaction and weight and shape concerns at age 14 years (n= 1,751, complete cases)

| **Outcomes** | **Exposure: third of IL-6** | | **Exposure: third of CRP** | |
| --- | --- | --- | --- | --- |
|  | **2^nd^ third (vs 1^st^)**  **Mean difference (95%CI) in outcome** | **3^rd^ third (vs 1^st^)**  **Mean difference (95%CI) in outcome** | **2^nd^ third (vs 1^st^)**  **Mean difference (95%CI) in outcome** | **3^rd^ third (vs 1^st^)**  **Mean difference (95%CI) in outcome** |
| **Body dissatisfaction** |  |  |  |  |
| *Univariable* | 1.23 (0.34, 2.32), p=0.0071 | 2.32 (1.41, 3.23), p<0.0001 | 2.36 (1.47, 3.25), p<0.0001 | 3.41 (2.52, 4.30), p<0.0001 |
| *Multivariable model1* | -0.18 (-1.00, 0.65), p=0.67 | -0.18 (-1.04, 0.68), p=0.68 | 0.65 (-0.19, 1.49), p=0.13 | -0.20 (-1.09, 0.70), p=0.67 |
| *Multivariable model2* | -0.20 (-1.02, 0.62), p=0.63 | -0.30 (-1.16, 0.55), p=0.49 | 0.69 (-0.14, 1.52), p=0.10 | -0.18 (-1.07, 0.72), p=0.69 |
| **Weight & shape concerns** |  |  |  |  |
| *Univariable* | 0.33 (0.12, 0.53), p=0.0017 | 0.57 (0.37, 0.78), p<0.0001 | 0.61 (0.41, 0.81), p<0.0001 | 0.77 (0.57, 0.97), p<0.0001 |
| *Multivariable model1* | 0.03 (-0.16, 0.21), p=0.79 | 0.05 (-0.15, 0.24), p=0.62 | 0.24 (0.05, 0.43), p=0.01 | 0.01 (-0.19, 0.22), p=0.91 |
| *Multivariable model2* | 0.01 (-0.17, 0.20), p=0.89 | 0.02 (-0.17, 0.22), p=0.83 | 0.25 (0.06, 0.44), p=0.01 | 0.03 (-0.18, 0.23), p=0.80 |

**Multivariable model 1**: adjusted for sex and total fat mass at age 9 years

**Multivariable model 2**: adjusted for sex, total fat mass at age 9 years, maternal education, maternal age at child’s birth, maternal pre-pregnancy BMI, maternal depression in pregnancy, mental health difficulties (i.e., total strength and difficulties questionnaire score) at age 7 years, peer victimization at age 8 years, maternal experience of stressful life events in pregnancy.

## References

1. Dencker M, Thorsson O, Lindén C, Wollmer P, Andersen LB, Karlsson MK (2007): BMI and objectively measured body fat and body fat distribution in prepubertal children. *Clin Physiol Funct Imaging*. doi: 10.1111/j.1475-097X.2007.00709.x.

2. Freedman DS, Wang J, Maynard LM, Thornton JC, Mei Z, Pierson RN, *et al.* (2005): Relation of BMI to fat and fat-free mass among children and adolescents. *Int J Obes*. doi: 10.1038/sj.ijo.0802735.

3. Goodman R (1997): The Strengths and Difficulties Questionnaire: a research note. *J Child Psychol Psychiatry*. 38: 581–6.

4. Wolke D, Woods S, Bloomfield L, Karstadt L (2000): The association between direct and relational bullying and behaviour problems among primary school children. *J Child Psychol Psychiatry Allied Discip*. doi: 10.1017/S0021963099006381.

5. Dorrington S, Zammit S, Asher L, Evans J, Heron J, Lewis G (2014): Perinatal maternal life events and psychotic experiences in children at twelve years in a birth cohort study. *Schizophr Res*. 152: 158–63.

6. Murray L, Carothers AD (1990): The validation of the Edinburgh Post-natal Depression Scale on a community sample. *Br J Psychiatry*. 157: 288–90.

7. Angold A, Costello EJ, Messer SC, Pickles A, Winder F, Silver D (1995): Development of a short questionnaire for use in epidemiological studies of depression in children and adolescents. *Int J Methods Psychiatr Res*. 5: 237–249.

8. Skuse DH, Mandy WPL, Scourfield J (2005): Measuring autistic traits: heritability, reliability and validity of the Social and Communication Disorders Checklist. *Br J Psychiatry*. 187: 568–572.

9. van Strien T, Frijters JER, Bergers GPA, Defares PB (1986): The Dutch Eating Behavior Questionnaire (DEBQ) for assessment of restrained, emotional, and external eating behavior. *Int J Eat Disord*. 5: 295–315.
